# Total Arsenic, pH, and Sulfate Are the Main Environmental Factors Affecting the Microbial Ecology of the Water and Sediments in Hulun Lake, China

**DOI:** 10.3389/fmicb.2020.548607

**Published:** 2020-09-24

**Authors:** Yongquan Shang, Xiaoyang Wu, Qinguo Wei, Huashan Dou, Xibao Wang, Jun Chen, Huanxin Zhang, Shengchao Ma, Honghai Zhang

**Affiliations:** ^1^College of Life Sciences, Qufu Normal University, Qufu, China; ^2^Hulunbuir Academy of Inland Lakes in Northern Cold & Arid Areas, Hulunbuir, China; ^3^College of Marine Life Sciences, Ocean University of China, Qingdao, China

**Keywords:** bacterial community structures, biogeochemical cycling processes, microbial community structures, water ecosystems, sediment ecosystems, Hulun Lake

## Abstract

Bacteria have the metabolic potential to produce a diverse array of secondary metabolites, which have important roles in biogeochemical cycling processes. However, for Hulun Lake and the rivers that enter into it, the bacterial community structures and their effects have not previously been widely studied, limiting our ecological understanding of this habitat. To address this, we have analyzed the bacterial communities in the water ecosystem of the Hulun Lake Basin. 16S rRNA high-throughput sequencing identified 64 phyla, 165 classes, 218 orders, 386 families, and 740 genera of bacteria across all samples. The dominant phyla in the central area of the lake were Proteobacteria, Actinobacteria, Firmicutes, and Cyanobacteria, while in all other areas, Proteobacteria, Actinobacteria, and Bacteroidetes were dominant. The microbial community structures were significantly affected by environmental factors [arsenic (As), pH, and sulfate (SO_4_^2–^)] and their location in the lake. The species richness in the sediments of Hulun Lake was higher than in the water, and this ecosystem harbored the highest proportion of unclassified sequences, representing unclassified bacteria. This study provides basic data for future investigations into the Hulun lake ecosystem and for water microbial monitoring and protection measures.

## Introduction

Inland lakes are important aquatic ecosystems that support complex animal and plant communities. In these ecosystems, microorganisms in the sediments and water make up the lowest levels of the food chains and drive biogeochemical cycles. Lakes are important not only for freshwater ecosystems, but also as part of the Earth’s hydrosphere system, which provides a rich array of freshwater resources for humans. In addition, in recent years, due to human activities, global carbon dioxide (CO_2_) concentrations and other greenhouse gases have been increasing, causing the Earth’s climate to warm. The warming and drying climate has consequently caused the water area of Hulun Lake wetland to shrink and the water levels to decrease, year by year (Cai et al., 2016). Over the past few years, the warming climate and overgrazing have resulted in high concentrations of organic matter pollution in Hulun Lake, including its sediments.

Microorganisms are known for their metabolic potential to produce diverse secondary metabolites (Tyc et al., 2017), and this partially explains why bacteria are important in lake ecosystems. Through their own physiological and biochemical reactions, they can promote the cycling of chemical elements in lakes, especially those of carbon, nitrogen, and phosphorus, and the exchange of nutrients, thus promoting the normal operations of the lake ecosystem (Gong et al., 2008). The habitat types vary greatly within and among lakes due to factors such as light intensity, lake water depth, pH, salinity, nutrition levels, chemical oxygen demand (COD), electrical conductivity, and oxygen content (Wu and Jiang, 2017). There is a large amount of variation in the number of microbial species in lakes and among the different habitat types. Kim et al. (2019) studied the community structures and diversity of the bacteria and fungi in soils with different salt levels using comprehensive evaluations of the soil characteristics and pyrophosphate sequencing techniques. It was found that electrical conductivity and salinity were the main factors affecting the structure and function of microbial communities in coastal reclamation areas (Kim et al., 2019). Zhang et al. (2019) studied the composition and dynamics of microbial communities in the Ganges Basin before and after the monsoon and rainy season using metagenomics technology. The α diversity and spatial heterogeneity of the microbial communities in the rainy season were found to be higher than those in the dry season. However, they weakened with increasing distance (Zhang et al., 2019). Wang W. et al. (2018) studied the differentiation of nitrogen and microbial communities between coastal and lacustrine sediments to explore the interactions between them. It was found that areas with lower nitrogen levels were correlated with a higher abundance and diversity of microorganisms, and these conditions increased around coastal zones. Restoring kelp, and its subsequent ecological functions, could also be very important for the treatment of eutrophic lakes (Wang W. et al., 2018).

Hulun Lake, as the largest lake in northern China, plays an important role in the protection of regional ecological environments. Previous studies have clearly shown that microorganisms play very important roles in lake ecosystems (Zhang et al., 2020) and in the cycling of carbon (Könneke et al., 2005; Quiza et al., 2014), nitrogen (van de Graaf et al., 1995; Stahl and de la Torre, 2012), phosphorus (P) (Hupfer and Lewandowski, 2008), and sulfur (Sorokin et al., 2011). As the ecological roles of the bacteria in Hulun Lake, including the sediments, are poorly understood, there is a need for their evaluation to better understand and protect the health of this ecosystem in the future.

The purpose of this study was to investigate the bacterial communities of the Hulun Lake Basin, with samples taken from water and sediments, to assess the relationships of the environmental factors such as P, pH, arsenic (As), and salinity, with the bacterial communities. This is the first report, to the best of our knowledge, of the microbial communities from Hulun Lake, China. This study will help to enhance the understanding of microbial populations and environmental variations and provide basic data for water microbial monitoring to protect Hulun Lake in the future.

## Materials and Methods

### Site Description and Sample Collection

Hulun Lake (117°00′10″–117°41′40″E, 48°30′40″–49°20′40″N) is the largest freshwater lake in northern China (Inner Mongolia Hulun Lake to national nature reserve annals) and also the largest freshwater lake in the central Asian grasslands. It is located in the Hulunbuir grassland of the Inner Mongolian autonomous region (Zhang et al., 2018). When the water level of Hulun Lake is 545.3 m, the water storage volume is ∼13.8 billion m^3^, the water surface area is ∼2,339 km^2^, the maximum water depth is 8 m, and the average water depth is 5.7 m (Inner Mongolia Hulun Lake to national nature reserve annals) (Wang et al., 2020). Overall, Hulun Lake is an irregular oblique long shape, showing a northeast southwest trend. Due to its arid continental monsoon climate, there is scarce rainfall throughout the year.

**FIGURE 1:**
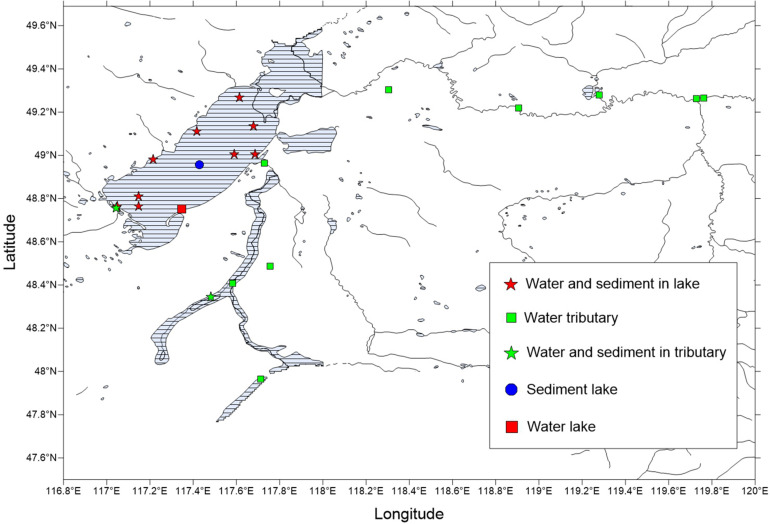
Distribution map of the winter sampling points in the Hulun Lake Reserve. The latitude and longitude of the sampling points are shown in Supplementary Table 1.

Preliminary investigation and demonstration were conducted prior to the sample collections for this study. Through the preliminary investigation, 21 sampling points were identified for the collection of water samples and sediments during the winter. The sample sites covered the entire lake and all tributaries or surrounding rivers and consisted of water and sediments that were taken from December 28, 2018, to December 29, 2018. However, there were no sediment samples for some locations. There were 21 water samples and 12 sediment samples selected for the physical and chemical analyses and sequencing (Figure 1 and Supplementary Table 1).

The water samples were sampled according to the depth of the upper and lower layers. The upper sampling position was 0.5 m from the depth of the water surface, and the lower sampling position was 0.5 m above the lake bottom. For some shallow sampling points (water depth ≤ 1 m), only one water sample was collected, at 0.5 m from the top. The collection device used consisted of a 2.5-L plexiglass water collector and a 10-L sterile polyethylene bucket. The sampling buckets were washed with ultra-pure water and sterilized with 70% alcohol before use. Before the sample was collected, the water from each sampling point was washed three times, then three barrels were collected, refrigerated in a special ice box, and quickly transported back to the laboratory for storage at −20°C for further use. At the same time, the physical and chemical indexes were measured. The collection of the sediments was carried out using a professional sediment grabber. Samples were quickly transported back to the laboratory and stored at −80°C for further use. Among the 21 water sampling points, WHL1–WHL10 were from lake water (WHL group) and WHL18–WHL28 were from water tributary (WHLHL group). Among the 12 sediment sampling points, WHLN1–WHLN10 were from Hulun Lake, and WHLN11–WHLN12 were sediment tributary. Water samples from the 21 collection points were divided into three groups, including three points in the lake center (WHLHZ group) (WHL8, WHL9, and WHL10), seven points around the lake (WHLHB group) (WHL1–WHL7), and the remaining points (WHL18–WHL28) were from the tributaries of the lake (WHLHL group). All water samples were in WZS groups, and all sediments samples were in WZN groups (Supplementary Table 1).

### Measurement of Environmental Factors

The physical and chemical indexes such as water temperature, pH, dissolved oxygen, and conductivity were determined using a three-channel analyzer (portable) (WTW, Germany), and the remaining environmental factors were determined in the laboratory according to standard methods (Fang et al., 2015; Han et al., 2020) (Supplementary Table 2).

### Sample Processing, DNA Extraction, and PCR Amplification

The water samples used for the microbiological analyses were sent back to the laboratory in a special ice box on the same day, and the water samples were filtered with a circulating water vacuum pump on a super-clean worktable, and the water samples were filtered through a 0.22-μm filter membrane. To ensure the reliability of the experimental results, three replications were made for each sample. The filtered membrane was preserved at −80°C. To avoid contamination, sterile techniques were used throughout all processes. According to the manufacturer’s protocols, microbial DNA was extracted using the HiPure Soil DNA Kits and HiPure Stool DNA Kits (Magen, China). We amplified the V3-V4 region of the bacterial 16S rRNA genes with the bacterial universal primers 341 F (CCTACGGGNGGCWGCAG) and 806 R (GGACTACHVGGGTATCTAAT). PCR reactions were performed in triplicate, with 50-μl mixtures containing 5 μl of 10 × KOD Buffer, 5 μl of 2.5 mM dNTPs, 1.5 μl of each primer (5 μM), 1 μl of KOD polymerase, and 100 ng of template DNA. The PCR amplification conditions were 95°C for 2 min, followed by 27 cycles at 98°C for 10 s, 62°C for 30 s, and 68°C for 30 s and a final extension at 68°C for 10 min.

### Illumina Hiseq 2500 Sequencing

Amplicons were extracted from 2% agarose gels and purified using the AxyPrep DNA Gel Extraction Kit (Axygen Biosciences, Union City, CA, United States), according to the manufacturer’s instructions and quantified using ABI Step One Plus Real-Time PCR System (Life Technologies, Foster City, CA, United States). Purified amplicons were pooled in equimolar and paired-end sequenced (PE250) on an Illumina platform (Illumina Hiseq 2500, United States) according to the standard protocols.

### Quality Control, Read Assembly, and Taxonomic Classification

To get high-quality clean reads, the raw reads were further filtered according to the following rules using FASTP^1^ : reads containing more than 10% unknown nucleotides (N) and less than 80% of bases with quality (Q-value) >20 were removed. Then, the paired-end clean reads were merged as raw tags using FLASH (Tanja and Salzberg, 2011) (version 1.2.11), with a minimum overlap of 10 bp and mismatch error rates of 2%. Noisy sequences in the raw tags were filtered by QIIME (version 1.9.1) (Caporaso et al., 2010). The clean tags were then searched for against the reference database^2^ to perform reference-based chimera checking using the UCHIME algorithm^3^. All effective tags were used for further analysis. The effective tags were clustered into operational taxonomic units (OTUs) of ≥97% similarity using the UPARSE (Edgar, 2013) pipeline. The tag sequences with the highest abundance were selected as representative sequences within each cluster. Rarefaction curves were created using Origin software (Supplementary Figure 1). The rarefaction curves were used to evaluate whether the sequencing quantity was sufficient to cover all groups and to indirectly reflect the richness of the species in the sample. The representative sequences were classified into organisms using a naive Bayesian model using an RDP classifier (version 2.2) (Wang et al., 2007) based on SILVA (Pruesse et al., 2007) Database^4^, with confidence threshold values ranging from 0.8 to 1. Biomarker features in each group were screened by Metastat (version 20090414) (White et al., 2009) and LEfSe software (version 1.0) (Segata et al., 2011). Linear discriminate analysis (LDA) effect size (LEfSe) (Segata et al., 2011) was used to select biomarkers in the WZS and WZN groups for the water and sediment samples. The threshold for the logarithmic LDA score for discriminative features was set at 2.0.

### Diversity Analysis

Chao1, Simpson, and all other alpha diversity indexes were calculated in QIIME, and the OTU rarefaction curves were also plotted in QIIME. Rarefaction curves were created using Origin software. The rarefaction curve was used to evaluate whether the sequencing quantity was sufficient to cover all groups and to indirectly reflect the richness of the species in the sample. Weighted and unweighted UniFrac distance matrices were generated by QIIME. Multivariate statistical techniques, including non-metric multidimensional scaling (NMDS) of unweighted UniFrac distances, were generated using R with the Vegan package (version 2.5.3) and plotted using the ggplot2 package (version 2.2.1) (Simpson et al., 2010). The Adonis (also called Permanova) and Anosim tests were conducted in R using the Vegan package (version 2.5.3) (Wickham and Chang, 2015). Non-parametric statistical analyses on the rarefied data were performed using “adonis” and “anosim” functions in R (Clarke, 1993).

### Analysis of the Relationships Between Environmental Factors and Microbial Communities

Canonical correspondence analysis (CCA) was used to reveal the relationships between the microbial communities and environmental factors (Braak, 1986). For this, the CCA functions in R using the vegan package were utilized. The “envfit” function (Dawson et al., 2012) with 999 permutations was used to reveal significant correlations between the environmental factors and microbial communities.

### Analysis of the Environmental Drivers of the Microbial Community Compositions

We based the relative abundance information for the species composition and functional composition data on the KEGG level 3 annotations, as well as the environmental factor information. The OTU classification and functional composition data were preprocessed, and the OTU with a relative abundance of less than 0.01% in any sample was removed. Then, the relative abundance was converted by log, and the value of each physicochemical data variable was converted into a z-score. Based on the Euclidean distance, the Mantel correlations (9999 permutations) between the physicochemical and structural data were calculated using the Mantel tests, and the corresponding taxonomic compositions and functions for each physicochemical property were then related to the wiring display. The results were determined in R (version 3.3.1) and then visualized in Adobe Illustrator (version 16.0.0) (Sunagawa et al., 2015).

### Co-occurrence Network Analysis

Networks were used to explore the co-occurrence patterns of the bacterial taxa. The data screening criteria were genera with relative abundances higher than 0.05% and Spearman correlation coefficients (*r*) >0.6, with *P* values <0.01 (Barberán et al., 2012). All strong correlations were identified using the paired comparisons of the dependent abundances, and then a correlation network was formed, where each node represents a genus and each line (edge) represents a node, i.e., a strong and significant correlation between the genera. Cytoscape was used to construct a diagram of the interaction networks. The iGraph package was used in the R environment to calculate a set of measures for the network [number of nodes and edges, average path length (APL), network diameter (ND), average degree (AD), graph density (GD), clustering coefficient (CC), and modularity (MD)] (Csardi and Nepusz, 2006). Meanwhile, 10,000 Erdõs–Rényi random networks were generated to compare with the topology of the real network, with each edge having the same probability of being assigned to any node (Erdõs and Rényi, 1961). The resulting module network graph showed module divisions based on the results of the function predictions and used different colors to distinguish the different modules.

## Results

### Environmental Parameters

The values of the environmental parameters in the lake and its tributaries are shown in Supplementary Table 2. From the environmental factors, Hulun Lake and its tributaries were determined to be weakly alkaline (pH: 7.96∼8.9), and there was a highly significant difference among the three sites (Kruskal–Wallis, *P* < 0.01). The range of the electrical conductivity was 821.9–1,917.3 μs/cm, and there was a highly significant difference among the three sites for this as well (Kruskal–Wallis, *P* < 0.01). The ammonia nitrogen content in the tributaries was higher than that in Hulun Lake, but there was no significant difference among the three tributatires (Kruskal–Wallis, *P* > 0.05). The total P, total nitrogen, fluoride, and total As contents were lower in the rivers entering the lake, and there were significant differences among the three (Kruskal–Wallis, *P* < 0.05). The pH and salinity of Hulun Lake were significantly higher than those of the rivers entering the lake (Kruskal–Wallis, *P* < 0.05).

### Microbial Compositions in the Hulun Lake Samples

After removing the low-quality sequences and mismatches, a total of 13,896,677 effective tags from the 33 sites were obtained. When the high-quality sequences at a level of 97% similarity were grouped, there were 211,298 OTUs formed, with a per sample average of 1,663 OTUs. The rarefaction curve shows that when the sequence numbers for the samples reach 40,000, the curve tends to be flat, indicating that the sequencing depth had covered all species in the sample, and reflected the community structure and diversity of the species to a certain extent (Supplementary Figure 1). We identified a total of 64 phyla (57 bacterial and seven archaea), 165 classes, 218 orders, 386 families, and 740 genera across all samples.

**FIGURE 2:**
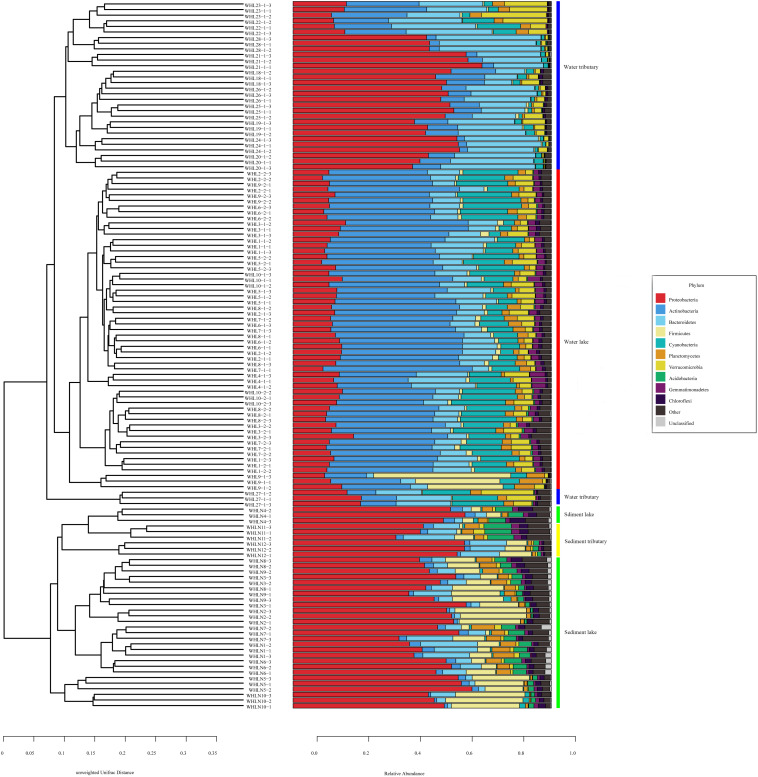
Unweighted pair group method with arithmetic mean (UPGMA) classification trees based on unweighted UniFrac at the phylum level (top 10). The colored vertical lines on the right of the bar chart indicate different sample types.

**FIGURE 3:**
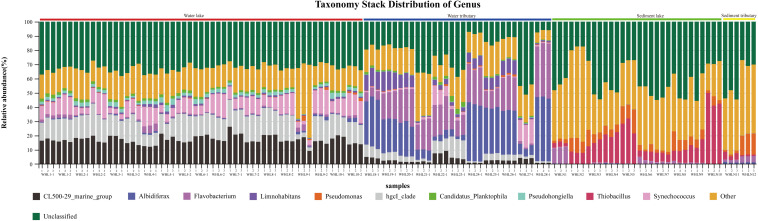
Taxonomy stack distribution at the genus level (top 10). There were three replicates per sample. The colored bars above the graph indicate different sample types.

In the water samples (WZS), the phylum with the largest relative abundance was Actinobacteria, accounting for ∼32.25% of the total sequences, followed by Proteobacteria (25.63%), Bacteroidetes (16.03%), Cyanobacteria (10.62%), Verrucomicrobia (5.05%), Planctomycetes (2.53%), Firmicutes (2.23%), Gemmatimonadetes (1.85%), and Parcubacteria (1.48%) (Figure 2). The above phyla were the dominant groups, and the relative abundances of the other phyla were less than 1%. At the genus level, there were 14 genera with relative abundances higher than 1%. *CL500-29_marine_group*, *hgcI_clade*, *Synechococcus*, *Albidiferax*, *Flavobacterium*, *Limnohabitans*, *Pseudohongiella*, *Candidatus_Planktophila*, *Candidatus_Limnoluna*, and *Fluviicola* were the top 10 genera with abundances of 17.80, 17.00, 11.14, 10.88, 9.51, 3.74, 2.27, 1.92, 1.91, and 1.76%, respectively (Figure 3). In the sediment samples (WZN), the phylum with the largest relative abundance was the Proteobacteria, accounting for about 58.32% of the total sequences, followed by Firmicutes (12.00%), Bacteroidetes (8.03%), Acidobacteria (4.20%), Planctomycetes (3.97%), Actinobacteria (2.94%), Chloroflexi (2.40%), and Gemmatimonadetes (1.20%) (Figure 2). Proteobacteria were overwhelmingly dominant in the sediment samples. The above phyla were the dominant groups, and the relative abundances of the other phyla were less than 1%. At the genus level, there were 13 genera with relative abundances higher than 1%; the top 10 of which were *Thiobacillus* (20.26%), *Pseudomonas* (10.82%), *Psychrobacter* (5.79%), *Massilia* (5.62%), *Bacillus* (4.29%), *Acinetobacter* (3.98%), *Flavobacterium* (3.97%), *Tumebacillus* (3.78%), *Rheinheimera* (3.22%), and *Hydrogenophaga* (2.14%) (Figure 3).

### Bacterial Alpha Diversity in the Sediment and Water

All the alpha diversity indexes for the samples are shown in Supplementary Table 3. The Good’s coverage was between 0.98 and 0.99, revealing that the sequencing results represented the true situation of the microflora structures. The Chao1 and Ace estimators for most sediment samples were higher than those of the water, which indicated that the species richness in the sediments was higher. In the water samples, we found that the Chao1 and Ace indexes in the lower lake water were higher than in the upper water of the lake and rivers, which also indicated that the species richness of the lower lake water was higher than that of the other two groups. Higher values for the Shannon and Simpson indexes indicated that the species richness and evenness in all the sediments (WZN groups) were higher than those in all the water (WZS groups) (Supplementary Figure 2).

### Community Compositions in Sediments Are More Similar Than Those in the Water

A non-parametric statistical test using “anosim” and “adonis” showed that the differences between the microbial communities in all of the eight groups were greater than the differences within the groups, which indicated that our grouping was still very reasonable (*P* < 0.01) (Table 1). The experimental results showed that there were significant differences among the eight groups, except for the two groups around the lake and the center of the lake (WHLHZ vs. WHLHB). At the same time, these tests showed that the maximum differences in the microbial community compositions were between the water and sediment groups (WZS vs. WZN).

**TABLE 1:** Statistical analysis of sediment and water samples from Hulun Lake using “adonis.”

Diffs	Df	Sums of sqs	Mean sqs	*F*-value	R2	*P*-value
WHLS vs. WHLX	1	0.84	0.84	7.31	0.12	0.001**
WHLS vs. WHLHL	1	1.56	1.56	11.03	0.15	0.001**
WHLHB vs. WHLHZ	1	0.15	0.15	1.16	0.02	0.171
NWH vs. NWHL	1	0.67	0.67	4.37	0.11	0.001**
WZS vs. WZN	1	7.52	7.52	45.58	0.27	0.001**
WHLX vs. WHLHL	1	2.05	2.05	14.46	0.20	0.001**
WHLS vs. WHLX vs. NWH	2	7.18	3.59	27.64	0.40	0.001**
WHLHB vs. WHLHZ vs. WHLHL	2	2.29	1.15	8.13	0.16	0.001**

**P < 0.01 adonis: Permutational multivariate analysis of variance.

**FIGURE 4:**
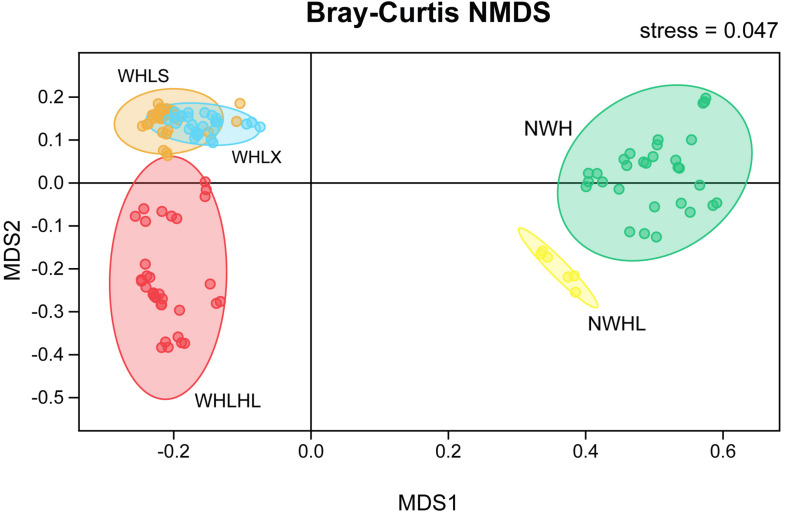
Non-metric multidimensional scaling (NMDS) ordination plot based on the Bray–Curtis dissimilarity data. Different colors represent different groups. Abbreviations: WHLS, lake water from the upper part of the lake; WHLX, lake water from the lower part of the lake; WHLHL, tributary water; NWH, sediment lake; NWHL, sediment tributary.

An NMDS ordination plot was produced based on the Bray–Curtis distance and showed that the sediments and water samples were far apart and that there was good distinction between them when compared with the sediment group, and the variability of the water group samples was greater (Figure 4). There was good distinction between the water samples of Hulun Lake and those of the river entering the lake, which shows that their microbiome community compositions were very different. However, the upper and lower samples of Hulun Lake were similar, indicating that their community compositions were also similar. The sediments at the lake bottom could gather with each other, but there was a distance between each point, which shows that the sediments at the lake bottom are still diverse, but that this diversity was smaller than that of the sediment tributary.

### Correlating Physicochemical Properties With Microbial Diversity

**FIGURE 5:**
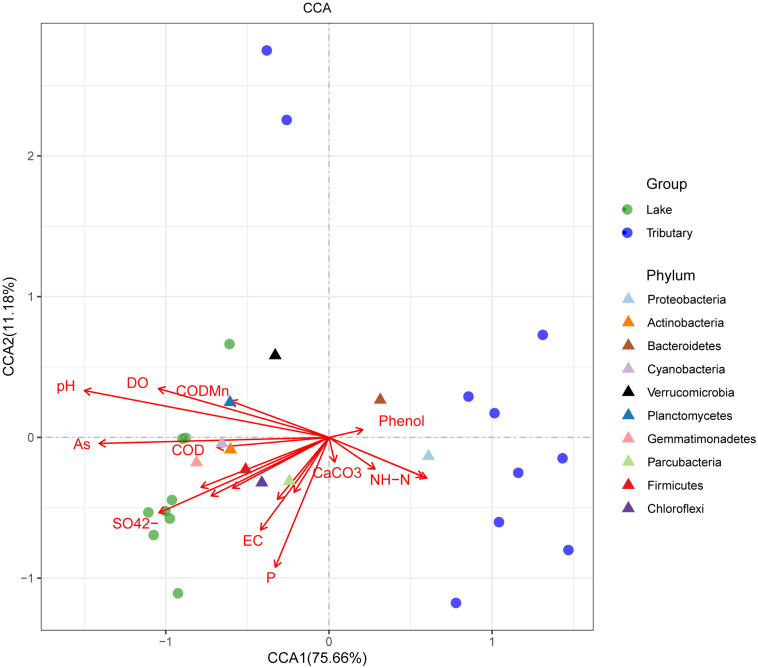
Canonical correlation analysis at the phylum level (by group). The length of the arrow of different environmental factors represents the degree of correlation between the corresponding environmental factor and the sample.

To explore the main reasons for the differences in the distributions of the water bacteria in Hulun Lake, redundancy analysis was used to analyze the structures of the water samples at the phylum level and the environmental physical and chemical indicators. Physicochemical properties, including CODMn, COD, salinity, mineralization, As, F-, pH, DO, sulfate (SO_4_^2–^), electrical conductivity, and temperature, were significant explanatory factors for the observed clustering pattern of the water microbial communities in the HLHB and HLHZ groups, while BOD5, phenol, CaCO_3_, and NH-N determined the water microbial community structures of the HLHL group, and the longer the arrow, the greater the influence on the distribution of the sample. From the diagram (Figure 5), we can see that pH, AS, SO_4_^2–^, and P were the main factors affecting the distribution of the samples. The relative abundance of the microflora in each sample can be judged by the distance between the microflora and each sample, and the closer the distance is, the higher the relative abundance is. The results showed that the distribution of the microorganisms in the center of the lake was the closest for all the samples. Compared with this, the distribution of the other sample points was obviously different. The relative abundance of the Proteobacteria and Bacteroidetes was higher in WHL18 and WHL19. The relative abundance of Gemmatimonadetes was higher in WHL2, WHL3, WHL6, and WHL7 (Supplementary Figure 3). Cyanobacteria, Verrucomicrobia, Chloroflexi, Actinobacteria, and Parcubacteria are close to each other in the map and close to the sampling point on the lake, indicating that the relative abundance of these bacteria is higher in the sampling point on the lake, but higher in the Proteobacteria and Bacteroidetes for the sampling point in the river. In addition, from the relationship between the environmental factors and species, the angle between pH and P is sharp, indicating that they are positively correlated with each other, and they are also positively correlated with Actinobacteria. However, Proteobacteria and Bacteroidetes with higher abundances were positively correlated with NH-N.

**FIGURE 6:**
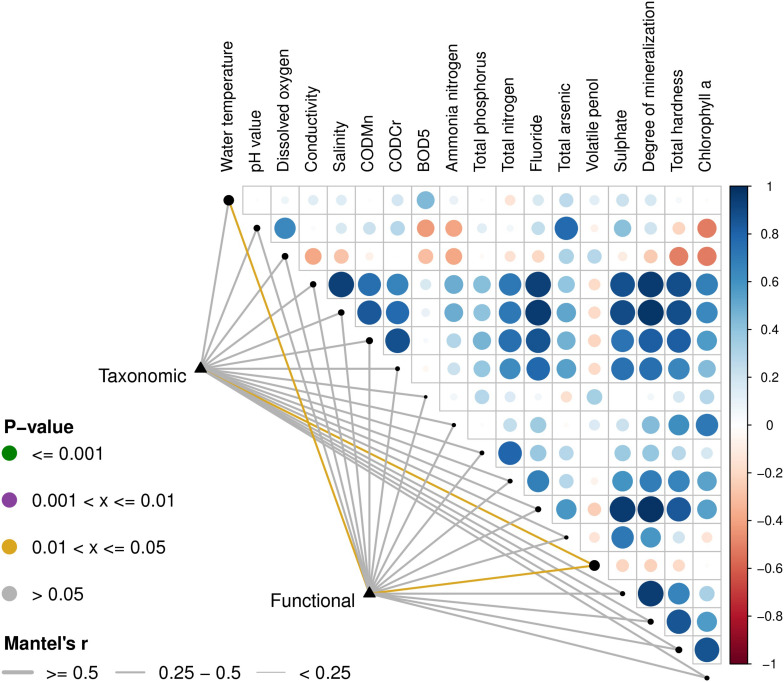
Environmental drivers of microbial community compositions in water. The distance correlation of the Mantel *r* statistics in the lower left corner corresponds to the line width, and the statistically significant *P* value corresponds to the line color. The upper right triangle is a display of the calculation for the Pearson correlation coefficient (PCC) between each physical and chemical factor, the circle color indicates the correlation coefficient on the corresponding color axis, and the circle size indicates the absolute value of the corresponding correlation coefficient. Therefore, the darker the color, the greater the correlation, the lighter the color, the weaker the correlation. The corresponding longitudinal circles of each environmental factor correspond to the pairwise correlations of the environmental factors arranged from far to near on the left side, respectively. The size of the black dots on the diagonal represents the size of the addition of the species composition data and the functional composition data of the Mantel’s *r*-statistics.

Based on the Euclidean distances from the Mantel test, the Mantel correlations between the physical and chemical data and the composition data were calculated, and the taxonomic composition and functional composition data were associated with each physical and chemical property, respectively. As can be seen in Figure 6, the physical and chemical properties of the water, including water temperature and volatile phenols, were closely related to classification and function.

### Biomarker Discovery

**FIGURE 7:**
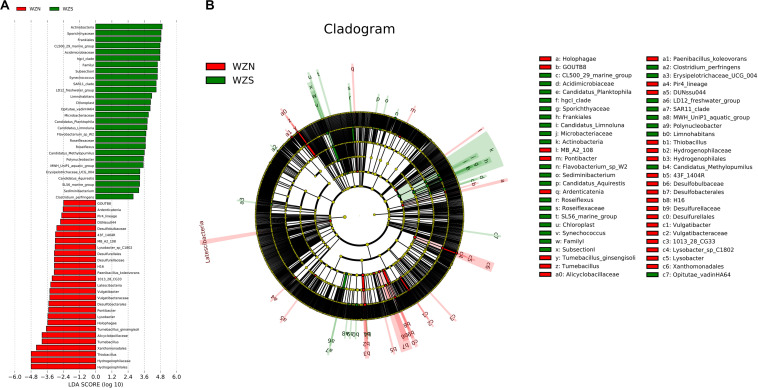
Biomarker analysis of the water and sediment microbial communities from the WZS and WZN. **(A)** Differentially abundant taxa, the length of the histogram represents the impact of the different species [linear discriminate analysis (LDA) = 2]. **(B)** Cladogram showing the phylogenetic structures of the microbiota. In the branching diagram of their evolution, the circles that radiate from inside to outside represent taxonomic levels from kingdom to species, and each small circle at a different taxonomic level represents a species at that taxonomic level. The diameter of the circles is proportional to the relative abundance. Species that are not significantly different are uniformly colored yellow.

In water and sediment samples, the LEfSe analysis identified 28 biomarkers for the WZS group and 27 for the WZN group (Figure 7). The most differentially abundant bacteria from the WZS group were the Actinobacteria, Sporichthyaceae, and Frankiales. These included members of the Acidimicrobiaceae and Microbacteriaceae. Biomarkers in the sediment from the WZN mainly comprised numbers of the order Hydrogenophilales, family Hydrogenophilaceae, and the genera *Thiobacillus* and *Tumebacillus*.

### Co-occurrence Network Analysis

**FIGURE 8:**
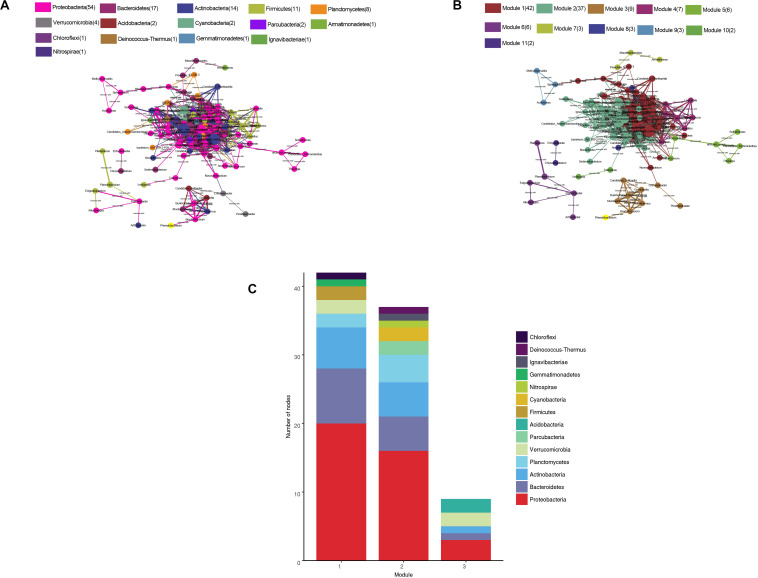
Network of co-occurring bacterial genera (relative abundance >0.05%) based on the correlation analysis. A connection represents a strong (Spearman’s *r* > 0.6) and significant (*P* < 0.01) correlation. The size of each node is proportional to the relative abundance; the thickness of each connection between two nodes (edge) is proportional to the value of the Spearman’s correlation coefficients. **(A)** Co-occurring network colored by phylum. **(B)** Co-occurring network colored by modularity class. **(C)** The first three modular species are composed.

The water microbial network consisted of 120 nodes (genera) and 938 edges (with a mean of 15.63 edges per node) (Figure 8). We compare the real network with the Erdõs and Rényi random network of the same size to illustrate the complex patterns and relationships between the nodes. The APL was 2.945 edges with a diameter of 5.7 edges. The CC was 0.622, and the modularity index (MD) was 0.256. Compared with the MDr (0.191), CCr (0.131), and APLr (1.98) of the Erdõs and Rényi random network, the structure of the real network was stronger. In general, the microbial networks at the genus level were closely related to each other, forming a small “topological world.”

All microbes in the network were assigned to 15 phyla. Among these, four phyla (Proteobacteria, Bacteroidetes, Actinobacteria, and Firmicutes) were widely distributed, accounting for 80% of all nodes. When the nodes were distributed and modularized, all the nodes were divided into three main modules. The Module I nodes mainly belonged to the Proteobacteria, Bacteroidetes, and Actinobacteria; module II nodes were mainly Proteobacteria, Bacteroidetes, Actinobacteria, and Planctomycetes; and module III nodes were mainly Proteobacteria, Verrucomicrobia, and Acidobacteria.

## Discussion

Chemical pesticides, fertilizers, cow and sheep feces, and other factors will not only have a negative impact on the physical and chemical properties of the lake water but also have an important impact on the species, quantities, community compositions, and distributions of microorganisms in the overlying water and sediments of Hulun Lake. The effects on the microbes are mostly negative. The change in the number of microbial species will react to the nutrient cycles of the lake water and the decomposition of the organic matter, thus forming a cycle process.

In this study, the overlying water and sediments of Hulun Lake and its rivers were sequenced and analyzed, and the communities and compositions of the microorganisms in Hulun Lake, and their interactions with environmental factors, were studied. We found that although there were no significant differences in the potassium permanganate index and COD among the three groups, their values were essentially of an inferior class V standard. There were significant differences in the pH and salinity among the three groups, and from a numerical point of view, the pH and salinity of Hulun Lake were significantly higher than those of the rivers entering the lake. The reason for this phenomenon may be due to the dry climate and the loss of water, leading to an increase in the various mineralization and ion concentrations in the lake, which in turn leads to an increase in pH and salinity. This change will also significantly affect the composition and distribution of microbial communities in Hulun Lake. As an important index of water eutrophication, total nitrogen and total P were also significantly smaller in the rivers entering the lake than those in Hulun Lake (*P* < 0.05). We speculate that, to some extent, this explains the high eutrophication index of Hulun Lake. The total P and total nitrogen levels may thus be caused by domestic sewage, and the fluctuating zones of people around the lake, resulting in the excrement from animals, such as cattle and sheep and hay from the falling zone, to enter Hulun Lake. However, this theory requires further investigation and experimental verification.

There have been many previous investigations into the differences in microbial diversity between sediment and water samples (Mesbah et al., 2007; Feng et al., 2009; Fang et al., 2015; Qu, 2015). For example, Feng et al. (2009) showed that in the Changjiang estuary and coastal area of the East China Sea, the Shannon–Weaver diversity index values indicate that bacterial diversity in the sediment samples was much higher than in the water samples (Feng et al., 2009). Our results are consistent with previous studies reporting that sediments have a higher Shannon’s diversity than water samples (Fang et al., 2015; Qu, 2015). Here, the ACE and Chao1 estimators for richness were also found to be higher for the sediment samples. This reflects the conclusion that the bacterial diversity of the Hulun Lake sediment samples was higher than that of the water samples.

Different environmental indicators have different effects on the structure of microphytic communities. Xiong et al. (2014) found that environmental and spatial variability can significantly affect the structures of bacterial communities (Xiong et al., 2014; Liang et al., 2016). Huang et al. (2018) found that OM, total phosphorus (TP), pH, and DO were the main factors affecting the bacterial communities at the mouth of Taihu Lake, while OM, DO, and pH were not the main environmental factors affecting the sediment communities there (Huang et al., 2018). Rubin and Leff (2007) found that inorganic nitrogen and soluble P in the water of the Ohio River, United States, had a large influence on the microbial community structures. Ammonia nitrogen, total nitrogen, nitrate nitrogen, and chlorophyll have the greatest influences on the distribution of phytoplankton communities in Dianchi Lake (Kent et al., 2003). The CCA diagram shows that the different environmental factors have different effects on the microbial communities. The pH and SO_4_^2–^ are the main environmental factors affecting the sample distributions and microbial communities, and the Proteobacteria had a great influence on the WHLHL grouping. From the correlation demonstration results, the temperature and phenol were seen to have significant effects on the species compositions and functional compositions of the bacteria. These results are quite different to those of previous investigations from Poyang Lake (Ding et al., 2015; Ren et al., 2019) and Honghu Lake (Han et al., 2019). Studies have shown that most Proteobacteria bacteria play an important role in nitrogen removal, biological P removal, and organic degradation (Nguyen et al., 2011). We speculated that the nitrogen and P contents in the WHLHL groups might be low under the action of microorganisms, and the measurement results from this investigation supported this conclusion. This may also be an important reason for the better water quality in this group. The differences and similarities among the samples in this study reflect the particularity of microbial community structures and show that they also have a far-reaching significance for understanding the properties and functions of microorganisms.

According to the distribution characteristics of the microbiota in the gate, the dominant bacteria for the three groups did not change greatly, but their abundances were quite different. In this study, the microbial communities in the central area of the lake were mainly concentrated in Proteobacteria, Actinobacteria, Firmicutes, and Cyanobacteria. The lake periphery and river areas were mainly concentrated in Proteobacteria, Actinobacteria, and Bacteroidetes. Similar results have been obtained in previous studies, as Shao et al. (2011) found that Proteobacteria and Chloroflei were the dominant sediment groups in Taihu Lake, Zaitseva et al. (2014) found that Proteobacteria, Firmicutes, and Bacteroidetes were the dominant phyla in Lake Beloe sediments, and Yu et al. (2014) found similar results for the saline Lake AWongco on the Tibetan Plateau. Bacteroidetes can degrade complex molecules into simple compounds under anaerobic conditions (Dai et al., 2013). In our study, Bacteroidetes accounted for about 10% of the total abundance. Studies have also shown that Bacteroidetes and Acidobacteria have little correlation with eutrophication, and that the abundance of Actinobacteria is high in regions with low eutrophication (Liu et al., 2009; Xue et al., 2018). Proteobacteria are widely distributed in a variety of environments and play an important role in the degradation of organic compounds. It has previously been pointed out that salinity is an important factor affecting the growth of Proteobacteria, and there were no Proteobacteria in marine environments and saline-alkali soils. In our study, the abundance of Proteobacteria and Actinobacteria was high, which indicates that the eutrophication and salinity of Hulun Lake were relatively low and was consistent with our measurement values. This further confirms the reliability of our experiment and shows that these microbes are also beneficial to the environment.

A non-parametric statistical test using “anosim” and “adonis” showed that for the WHLHZ and WHLHB groups, there was no significant difference (*P* = 0.171 > 0.05). We speculated that the reason for this phenomenon may be better in the interior mobility of Hulun Lake in winter. While the weather is cold in the winter and the ice layer on the surface of the lake is very thick, there may be a flow phenomenon below the ice layer. This flow covered the whole lake, resulting in no significant differences between the microbial communities at the center of the lake and the area around the lake.

As biomarkers in the water samples, Acidimicrobiaceae are known to play key roles in the Feammox process (ammonium oxidation coupled to iron reduction), and Microbacteriaceae that are widely distributed in marine, terrestrial, and freshwater environments are found to grow under very extreme conditions (Shuai and Jaffe, 2019), as they are heterotrophic, obligate aerobes (Pitt et al., 2019). For the biomarkers in the sediment samples, *Thiobacillus* is mostly known for its ability to oxidize sulfur compounds aerobically (Cardenas et al., 2010).

In our study, Proteobacteria, Bacteroidetes, and Actinobacteria were dominant in the bacterial communities, and they may play a key role in the structure and function of ecological communities. Shao et al. (2013) found that Proteobacteria gates were known to metabolize soluble organic substrates. Methylotenera are Proteobacteria that function as mandatory methyl utilization agents (Kalyuzhnaya et al., 2006). Studies have found that Bacteroides, represented by Mucilagiginibacter, play an important role in the degradation of various biopolymers (Pankratov et al., 2007). Acidobacter also plays an important role in polymer degradation and other aspects, including *Granulicella* as a representative (Pankratov and Dedysh, 2010). Lillington et al. (2020) found that anaerobic microbial communities can degrade and promote the carbon cycle on Earth.

In the WHLHL group, we found that there was a much higher abundance of *Flavobacterium* than in the WHL group and the WZN group. It has previously been documented that *Flavobacterium* plays an important role in the degradation of heavy metals (Al-Dhabi et al., 2019) and organic pollutants (Wan et al., 2019), which we speculate may be an important reason for the better water quality of the WHLHL group. *Thiobacillus* microbes differ greatly in their abundance, which are also much more abundant in sediments than in water samples. Research has shown that *Thiobacillus* is a member of the Beta-proteobacteria and is mostly known for its ability to oxidize sulfur compounds aerobically (Cardenas et al., 2010). We speculate that this may be related to the presence of more sulfides in the sediments. *Pseudomonas* plays an important role in processing heavy metals (Zhang et al., 2017; Wang X. et al., 2018). These microorganisms also play an important ecological role in our co-occurrence network.

The co-occurrence network analysis can explore the potential interactions between microbial communities and contribute to the interpretation of the structures of the complex microbial communities across spatiotemporal gradients (Barberán et al., 2012). By analyzing the big data of the Earth Microbiome Project, Ma et al. (2020) constructed a global microbiome coexistence network, and revealed the interconnection patterns among the microbiomes in various environments of the Earth, through the analysis of their “social relations.” Jiao et al. (2016) proved that the non-random symbiosis and connectivity of the bacterial communities in oil-contaminated oil fields using co-occurrence networks and explained the role of deterministic processes in the structure of these communities. By comparing our microbial network with the random network, it has proved that the microbial community in Hulun Lake and its rivers were non-random and connected, which indicates the role of the microorganisms in the community structures. In addition, based on the modular structures, the node was found to be mainly divided into three modules. The different modules mainly drive different functions (Newman, 2006). In network module I, some bacteria were associated with salt tolerance and phosphate tolerance. A lot of studies have shown that Albidiferax may have a good salt tolerance (Kaden et al., 2014). Studies have also shown that Gemmatimonas was related to the accumulation of phosphate (Pankratov and Dedysh, 2010). The main taxa in module II may be involved in electron transfer. For example, the iron-reducing bacteria *Thiobacillus*, sulfur-oxidizing bacteria *Sulfuritalea*, and the iron-oxidizing bacteria *Gallionella* may play a key role in electron migration (Hallbeck et al., 1993; Fortin et al., 1996). Some bacteria in module II were also involved in the degradation of organic pollutants, including Methylotenera and Rhodobacter. Bioelectron transfers play an important role in the degradation of organic matter (Stams et al., 2006). Some bacteria in module III are involved in the degradation of some polymers, including *Granulicella*, *Phenylobacterium*, and *Mucilaginibacter* (Lingens et al., 1985; Pankratov et al., 2007; Pankratov and Dedysh, 2010). Therefore, the community structure and functional processes and the community symbiosis model of the lake system are often non-random and function driven.

## Conclusion

This study revealed the effects of temperature, pH, AS, TP, DO, and other environmental factors on the compositions and distributions of the microbial communities in Hulun Lake and the rivers entering the lake. We found that As, pH, and SO_4_^2–^ were important environmental factors affecting the composition and distribution of microbial communities in Hulun Lake Basin. Compared with the overlying water, the species richness in the sediments of Hulun Lake was higher. The microbial communities in the central area of the lake were mainly concentrated with Proteobacteria, Actinobacteria, Firmicutes, and Cyanobacteria, while the microbial compositions in the other areas were mainly concentrated with Proteobacteria, Actinobacteria, and Bacteroidetes. In addition, in the overlying waters and sediments, we identified 28 biomarkers and 27 biomarkers, respectively, and detected many unclassified bacteria. Finally, co-occurrence networks showed that the microorganisms in Hulun Lake and its rivers are closely related and drive different ecological functions. Our research provides basic data for microbial monitoring and protections of Hulun Lake. In future work, it will be necessary to consider both temporal and spatial sampling and supplement the physical and chemical information and climatic information of the sediments. To better describe the temporal and spatial distributions of the community and functional structures, we need to combine them with the functional data for the microorganisms.

## Data Availability Statement

The datasets generated for this study can be found in the SRA database of NCBI, SRA accession: PRJNA613767 (https://www.ncbi.nlm.nih.gov/bioproject/PRJNA613767).

## Author Contributions

HHZ and YQS conceived and designed the study. YQS, XYW, QGW, HSD, XBW, and SCM performed the research. YQS, XYW, JC, and HXZ analyzed the data. YQS and XYW prepared the manuscript. All authors read and approved the final manuscript.

## Conflict of Interest

The authors declare that the research was conducted in the absence of any commercial or financial relationships that could be construed as a potential conflict of interest.

## Footnotes

1 https://github.com/OpenGene/fastp

2 http://drive5.com/uchime/uchime_download.html

3 http://www.drive5.com/usearch/manual/uchime_algo.html

4 https://www.arb-silva.de/
