# Supplementary material for: Total Arsenic, pH, and Sulfate Are the Main Environmental Factors Affecting the Microbial Ecology of the Water and Sediments in Hulun Lake, China
Source: Front Microbiol. 2020 Sep 24;11:548607. doi: 10.3389/fmicb.2020.548607 (PMC7541820; doi:10.3389/fmicb.2020.548607)
Supplement: Supplementary file 1 [file Data_Sheet_1.docx]

Supplementary Material

# Supplementary Figures and Tables

**Figure S1. Rarefaction curves of the OTUs across 126 samples from Hulun Lake. There are three replicates per sample**

**Figure S2. The box plot shows the alpha diversity of the different groups. WZS: all water samples WZN: all sediments samples**

**Figure S3. Canonical correlation analysis at the phylum level (by sample). The length of the arrow of different environmental factors represents the degree of correlation between the corresponding environmental factor and the sample**

**Table S1.** Latitude and longitude of the sampling points in Hulun Lake Reserve, China.

| Sampling location | Latitude | Longitude | Group | Sampling location | Latitude | Longitude | Group |
| --- | --- | --- | --- | --- | --- | --- | --- |
| WHLN1 | 49.269354 | 117.615023 | NWH | WHL1 | 49.269354 | 117.615023 | WHLHB |
| WHLN2 | 49.113955 | 117.417771 | NWH | WHL2 | 49.113955 | 117.417771 | WHLHB |
| WHLN3 | 48.982650 | 117.214470 | NWH | WHL3 | 48.982650 | 117.214470 | WHLHB |
| WHLN4 | 48.765062 | 117.048714 | NWH | WHL4 | 48.765062 | 117.048714 | WHLHB |
| WHLN5 | 48.766358 | 117.147589 | NWH | WHL5 | 48.766358 | 117.147589 | WHLHB |
| WHLN6 | 49.005982 | 117.685518 | NWH | WHL6 | 49.005982 | 117.685518 | WHLHB |
| WHLN7 | 49.138073 | 117.677915 | NWH | WHL7 | 49.138073 | 117.677915 | WHLHB |
| WHLN8 | 49.008014 | 117.590216 | NWH | WHL8 | 49.008014 | 117.590216 | WHLHZ |
| WHLN9 | 48.956750 | 117.428560 | NWH | WHL9 | 48.752050 | 117.347720 | WHLHZ |
| WHLN10 | 48.811069 | 117.147589 | NWH | WHL10 | 48.811069 | 117.147589 | WHLHZ |
| WHLN11 | 48.758878 | 117.044030 | NWHL | WHL18 | 49.304200 | 118.304200 | WHLHL |
| WHLN12 | 48.346360 | 117.481030 | NWHL | WHL19 | 48.486480 | 117.754970 | WHLHL |
|  |  |  |  | WHL20 | 48.758878 | 117.044030 | WHLHL |
|  |  |  |  | WHL21 | 48.346360 | 117.481030 | WHLHL |
|  |  |  |  | WHL22 | 47.964750 | 117.713470 | WHLHL |
|  |  |  |  | WHL23 | 48.964549 | 117.729684 | WHLHL |
|  |  |  |  | WHL24 | 49.264480 | 119.761170 | WHLHL |
|  |  |  |  | WHL25 | 49.261010 | 119.729150 | WHLHL |
|  |  |  |  | WHL26 | 49.217950 | 118.905520 | WHLHL |
|  |  |  |  | WHL27 | 49.279160 | 119.279170 | WHLHL |
|  |  |  |  | WHL28 | 48.408480 | 117.583240 | WHLHL |
|  |  |  |  |  |  |  |  |

**Table S2.** Kruskal-Wallis test of the physicochemical parameters of the water within Hulun Lake.

| Parameter | WHLHB | WHLHL | WHLHZ | p-value |
| --- | --- | --- | --- | --- |
| Temp (°C) | 0.10 ± 1.27 | -0.16 ± 0.28 | 0.87 ± 2.11 | 0.99 |
| pH | 8.90 ± 0.08 | 7.96 ± 0.54 | 8.73 ± 0.21 | 0.01^**^ |
| DO (mg/L) | 14.60 ± 0.97 | 12.27 ± 4.88 | 15.40 ± 0.10 | 0.24 |
| EC (μs/cm) | 1846.29 ± 103.93 | 821.90 ± 1435.44 | 1917.33 ± 29.84 | 0.01^**^ |
| Salinity (‰) | 0.80 ± 0.12 | 0.35 ± 0.63 | 0.87 ± 0.06 | 0.01^*^ |
| CODMn (mg/L) | 17.97 ± 2.06 | 13.29 ± 10.83 | 20.17 ± 4.08 | 0.29 |
| COD (mg/L) | 168.71 ± 46.91 | 101.80 ± 117.34 | 221.00 ± 7.00 | 0.19 |
| BOD_5_ (mg/L) | 3.96 ± 4.15 | 5.73 ± 3.25 | 6.77 ± 4.79 | 0.26 |
| NH^-N^ (mg/L) | 0.11 ± 0.04 | 0.13 ± 0.07 | 0.13 ± 0.08 | 1.00 |
| P (mg/L) | 0.12 ± 0.05 | 0.07 ± 0.10 | 0.37 ± 0.40 | 0.04^*^ |
| N (mg/L) | 2.06 ± 0.94 | 1.53 ± 1.66 | 3.55 ± 2.51 | 0.01^**^ |
| F^-^ (mg/L) | 2.79 ± 0.33 | 1.29 ± 1.57 | 2.76 ± 0.22 | 0.01^*^ |
| As (ug/L) | 42.19 ± 5.14 | 7.83 ± 12.55 | 29.90 ± 18.09 | 0.00^**^ |
| Phenol (mg/L) | 0.0003 ± 0.00 | 0.0004 ± 0.0003 | 0.0005 ± 0.0002 | 0.04^*^ |
| SO_4_^2-^(mg/L) | 1500.00 ± 20.73 | 57.15 ± 56.96 | 146.00 ± 16.09 | 0.01^*^ |
| mineralizatiom (mg/L) | 1084.00 ± 144.28 | 505.80 ± 615.79 | 1073.33±83.98 | 0.01^*^ |
| CaCO_3_ (mg/L) | 3.38 ± 0.26 | 2.93 ± 3.44 | 3.11 ± 0.23 | 0.16 |
| Chl (μg/L) | 4.86 ± 3.63 | 10.80 ± 20.75 | 3.67 ± 2.08 | 0.62 |

Note: Values shown were the average ± standard deviation from three different groups. WHLHB, WHLHL, WHLHZ is three different groups. The specific grouping is shown in Table 1. P^**^＜0.01； P^*^＜0.05 Temp, temperature; DO, dissolved oxygen; EC, conductivity; Salinity, salinity; NH^-N^, ammonia nitrogen; P, total phosphorus; N, total nitrogen; F^-^, fluoride; As, total arsenic; Phenol, volatile penol; SO_4_^2-^, sulphate; Mineralization, degree of mineralization; CaCO_3_, total hardness; Chl, chlorophyll a;

**Table S3.** Richness and evenness of the samples from Hulun Lake

| Samples | chao1 | Ace | observed_species | Shannon |
| --- | --- | --- | --- | --- |
| WHL1-1 | 1759.19 ± 48.51 | 1801.19 ± 58.95 | 1225.33 ± 0.47 | 6.25 ± 0.05 |
| WHL1-2 | 2217.45 ± 130.42 | 2233.07 ± 125.23 | 1678.66 ± 62.94 | 6.52 ± 0.09 |
| WHL2-1 | 1494.99 ± 218.87 | 1473.63 ± 231.06 | 1111.66 ± 113.74 | 6.52 ± 0.01 |
| WHL2-2 | 1944.59 ± 25.18 | 1987.26 ± 25.06 | 1403.00 ± 64.02 | 6.14 ± 0.10 |
| WHL3-1 | 1846.21 ± 140.38 | 1882.77 ± 146.27 | 1412.00 ± 97.96 | 6.72 ± 0.22 |
| WHL3-2 | 2539.12 ± 154.41 | 2518.40 ± 138.00 | 1963.00 ± 93.47 | 6.86 ± 0.10 |
| WHL4-1 | 1736.87 ± 126.86 | 1726.85 ± 102.34 | 1102.33 ± 24.00 | 6.34 ± 0.07 |
| WHL5-1 | 1585.41 ± 81.71 | 1600.03 ± 54.38 | 1075.00 ± 22.27 | 6.33 ± 0.03 |
| WHL5-2 | 1527.55 ± 106.57 | 1512.95 ± 72.76 | 1032.00 ± 36.71 | 6.20 ± 0.07 |
| WHL6-1 | 1467.80 ± 43.19 | 1447.22 ± 63.99 | 1077.00 ± 19.15 | 6.41 ± 0.04 |
| WHL6-2 | 1705.55 ± 137.19 | 1725.54 ± 135.83 | 1285.33 ± 56.44 | 6.18 ± 0.00 |
| WHL7-1 | 1365.76 ± 83.51 | 1314.65 ± 62.07 | 1006.33 ± 43.01 | 6.24 ± 0.24 |
| WHL7-2 | 2625.33 ± 169.46 | 2620.34 ± 146.43 | 1912.33 ± 70.78 | 6.61 ± 0.03 |
| WHL8-1 | 1353.46 ± 84.70 | 1332.41 ± 104.36 | 1031.00 ± 56.34 | 6.24 ± 0.01 |
| WHL8-2 | 2416.55 ± 76.32 | 2432.54 ± 77.77 | 1853.66 ± 78.50 | 6.48 ± 0.14 |
| WHL9-1 | 1168.59 ± 83.11 | 1120.89 ± 92.58 | 837.66 ± 72.61 | 5.58 ± 0.52 |
| WHL9-2 | 2036.70 ± 86.41 | 2073.17 ± 87.02 | 1455.33 ± 15.56 | 6.13 ± 0.01 |
| WHL10-1 | 1670.62 ± 96.10 | 1681.62 ± 97.20 | 1165.66 ± 30.66 | 6.22 ± 0.04 |
| WHL10-2 | 2202.13 ± 67.19 | 2190.09 ± 81.12 | 1695.33 ± 75.79 | 6.58 ± 0.07 |
| WHL18-1 | 1740.73 ± 66.60 | 1755.78 ± 43.61 | 1170.00 ± 70.44 | 5.37 ± 0.19 |
| WHL19-1 | 1559.13 ± 51.05 | 1504.66 ± 15.67 | 1031.66 ± 7.58 | 5.56 ± 0.02 |
| WHL20-1 | 1791.20 ± 77.67 | 1831.24 ± 73.44 | 1105.33 ± 18.03 | 5.22 ± 0.04 |
| WHL21-1 | 1298.29 ± 124.96 | 1265.39 ± 150.26 | 713.33 ± 33.82 | 4.37 ± 0.07 |
| WHL22-1 | 1380.84 ± 83.41 | 1338.04 ± 49.59 | 905.33 ± 15.53 | 6.05 ± 0.06 |
| WHL23-1 | 1389.77 ± 102.14 | 1360.16 ± 91.07 | 974.66 ± 39.55 | 6.61 ± 0.03 |
| WHL24-1 | 1288.19 ± 206.03 | 1315.03 ± 194.22 | 810.33 ± 101.02 | 4.19 ± 0.11 |
| WHL25-1 | 1876.89 ± 103.62 | 1905.70 ± 142.96 | 1224.66 ± 30.08 | 5.41 ± 0.06 |
| WHL26-1 | 1636.50 ± 94.35 | 1679.95 ± 56.57 | 995.66 ± 34.58 | 5.03 ± 0.04 |
| WHL27-1 | 2611.10 ± 120.61 | 2626.60 ± 86.40 | 1892.00 ± 121.79 | 6.72 ± 0.07 |
| WHL28-1 | 1696.43 ± 45.63 | 1705.81 ± 32.18 | 1023.66 ± 26.27 | 3.52 ± 0.09 |
| WHLN1 | 4051.33 ± 133.22 | 3942.50 ± 122.09 | 3423.00 ± 64.09 | 8.12 ± 0.20 |
| WHLN2 | 2531.05 ± 8.70 | 2442.48 ± 52.35 | 1954.33 ± 31.65 | 5.85 ± 0.03 |
| WHLN3 | 3331.62 ± 445.52 | 3252.57 ± 437.14 | 2828.33 ± 454.25 | 7.17 ± 0.80 |
| WHLN4 | 4087.52 ± 192.89 | 3973.92 ± 171.25 | 3591.66 ± 137.23 | 8.26 ± 0.17 |
| WHLN5 | 2336.95 ± 298.87 | 2241.91 ± 350.42 | 1710.66 ± 271.21 | 6.18 ± 0.16 |
| WHLN6 | 4124.33 ± 251.74 | 4025.21 ± 238.51 | 3443.66 ± 248.37 | 7.94 ± 0.25 |
| WHLN7 | 4267.08 ± 387.62 | 4190.79 ± 341.94 | 3524.33 ± 376.88 | 8.45 ± 0.22 |
| WHLN8 | 2966.61 ± 188.61 | 2845.06 ± 277.03 | 2353.33 ± 261.96 | 7.81 ± 0.32 |
| WHLN9 | 2922.13 ± 165.55 | 2791.26 ± 168.98 | 2265.00 ± 227.54 | 7.67 ± 0.49 |
| WHLN10 | 2243.72 ± 155.09 | 2118.83 ± 133.05 | 1525.00 ± 70.87 | 5.15 ± 0.25 |
| WHLN11 | 4016.27 ± 175.95 | 3901.58 ± 177.65 | 3464.33 ± 146.90 | 9.35 ± 0.01 |
| WHLN12 | 3271.85 ± 202.35 | 3122.43 ± 196.37 | 2625.33 ± 161.22 | 8.00 ± 0.13 |

Note: Values shown were the average ± standard deviation
