# Supplementary figures and images for: Total Arsenic, pH, and Sulfate Are the Main Environmental Factors Affecting the Microbial Ecology of the Water and Sediments in Hulun Lake, China
Source: Front Microbiol. 2020 Sep 24;11:548607. doi: 10.3389/fmicb.2020.548607 (PMC7541820; doi:10.3389/fmicb.2020.548607)

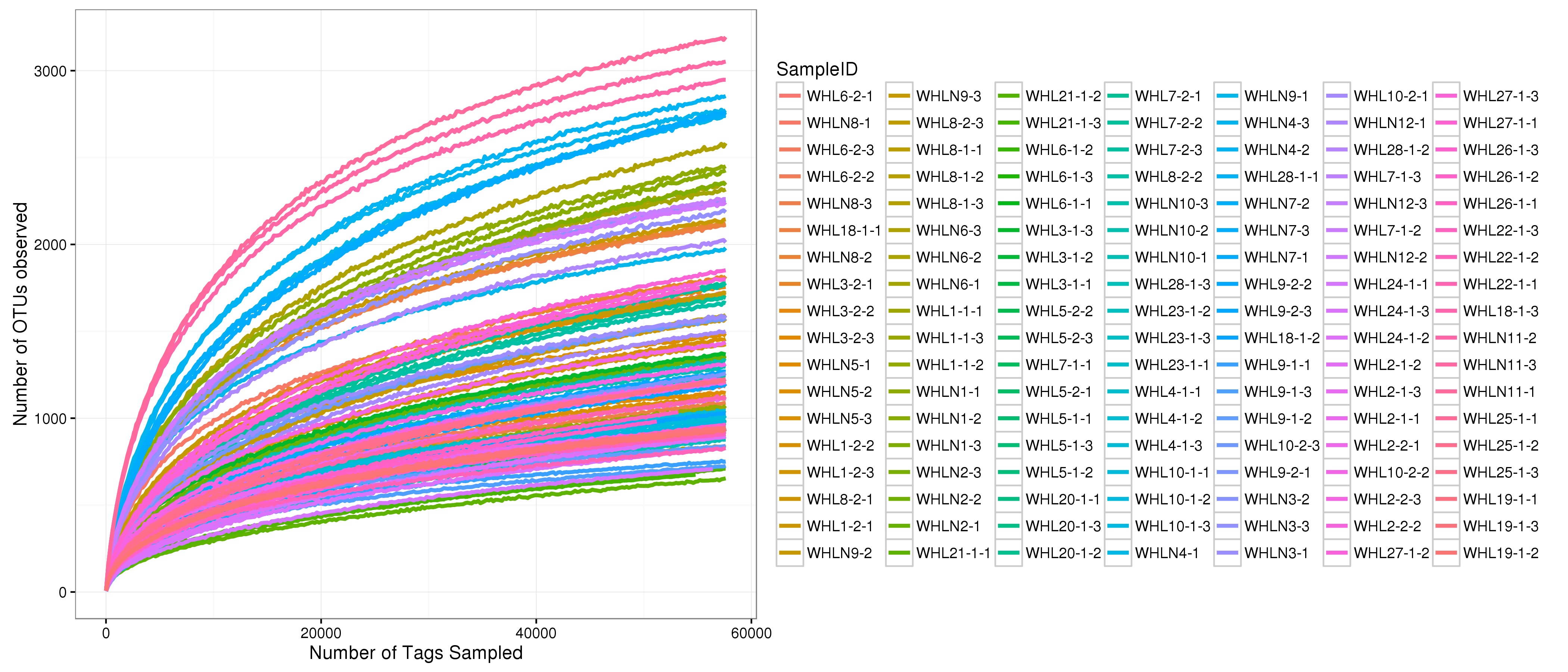

Supplement: Supplementary file 2 [file Image_1.JPEG]

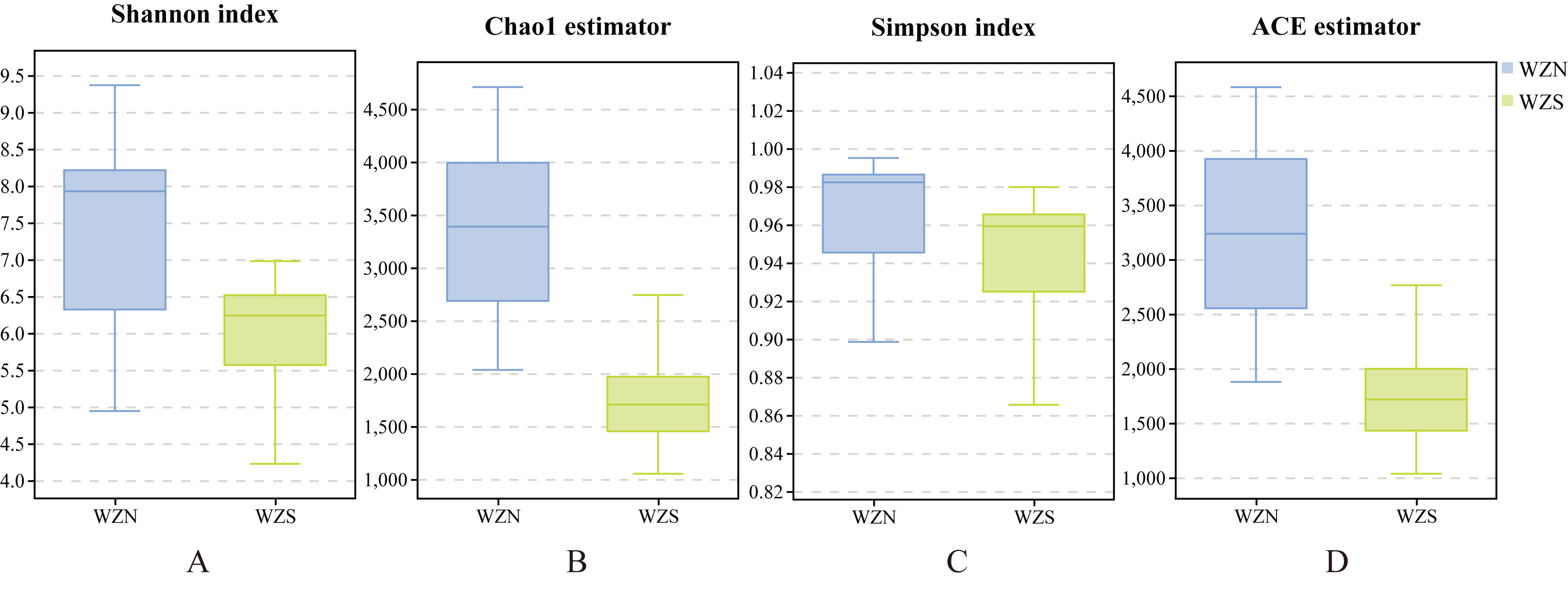

Supplement: Supplementary file 3 [file Image_2.JPEG]

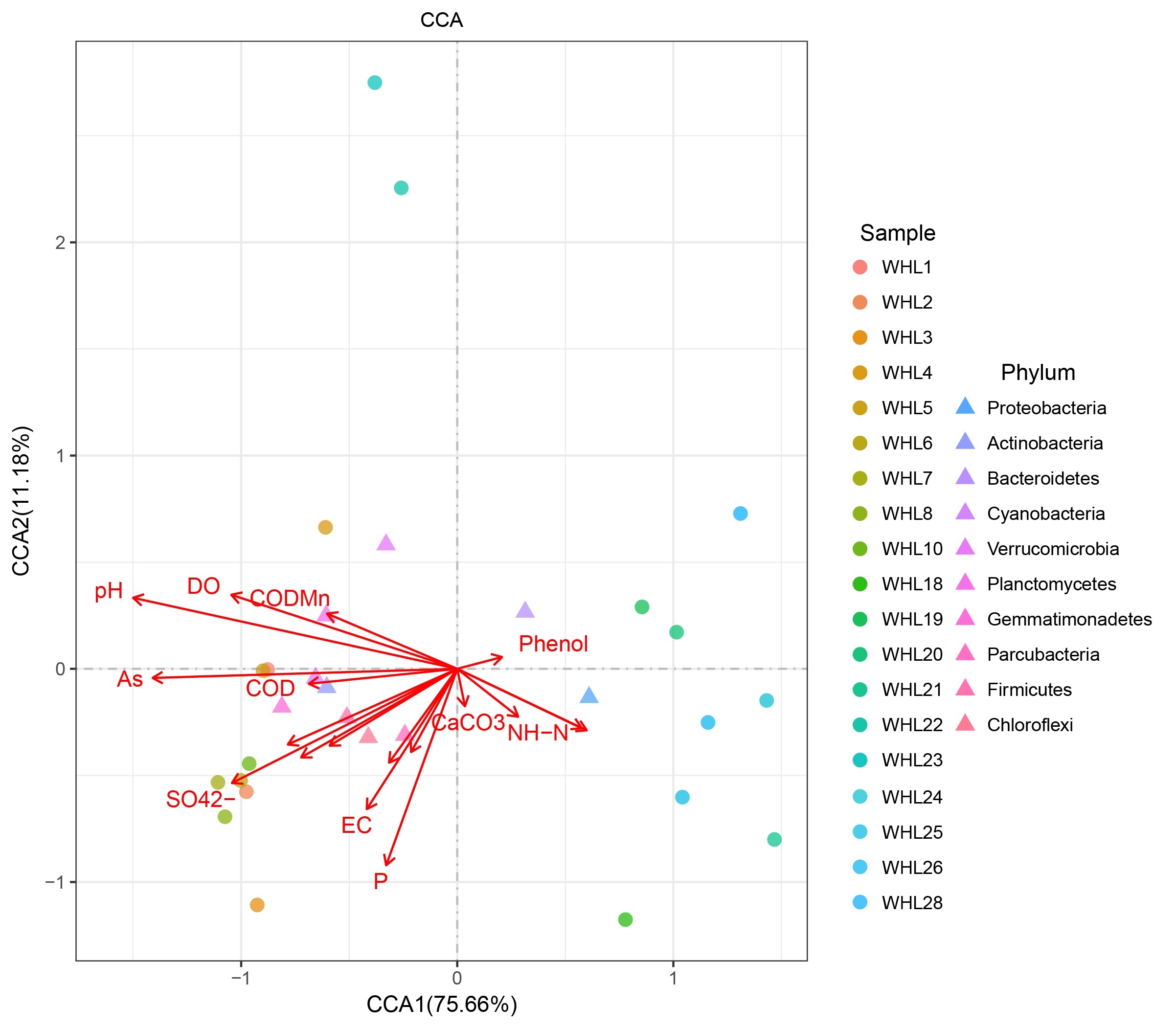

Supplement: Supplementary file 4 [file Image_3.JPEG]
